# Synergistic and Additive Effects of Hydrostatic Pressure and Growth Factors on Tissue Formation

**DOI:** 10.1371/journal.pone.0002341

**Published:** 2008-06-04

**Authors:** Benjamin D. Elder, Kyriacos A. Athanasiou

**Affiliations:** Department of Bioengineering, Rice University, Houston, Texas, United States of America; Tufts University, United States of America

## Abstract

**Background:**

Hydrostatic pressure (HP) is a significant factor in the function of many tissues, including cartilage, knee meniscus, temporomandibular joint disc, intervertebral disc, bone, bladder, and vasculature. Though studies have been performed in assessing the role of HP in tissue biochemistry, to the best of our knowledge, no studies have demonstrated enhanced mechanical properties from HP application in any tissue.

**Methodology/Principal Findings:**

The objective of this study was to determine the effects of hydrostatic pressure (HP), with and without growth factors, on the biomechanical and biochemical properties of engineered articular cartilage constructs, using a two-phased approach. In phase I, a 3×3 full-factorial design of HP magnitude (1, 5, 10 MPa) and frequency (0, 0.1, 1 Hz) was used, and the best two treatments were selected for use in phase II. Static HP at 5 MPa and 10 MPa resulted in significant 95% and 96% increases, respectively, in aggregate modulus (H_A_), with corresponding increases in GAG content. These regimens also resulted in significant 101% and 92% increases in Young's modulus (E_Y_), with corresponding increases in collagen content. Phase II employed a 3×3 full-factorial design of HP (no HP, 5 MPa static, 10 MPa static) and growth factor application (no GF, BMP-2+IGF-I, TGF-β1). The combination of 10 MPa static HP and TGF-β1 treatment had an additive effect on both H_A_ and E_Y_, as well as a synergistic effect on collagen content. This group demonstrated a 164% increase in H_A_, a 231% increase in E_Y_, an 85% increase in GAG/wet weight (WW), and a 173% increase in collagen/WW, relative to control.

**Conclusions/Significance:**

To our knowledge, this is the first study to demonstrate increases in the biomechanical properties of tissue from pure HP application, using a cartilage model. Furthermore, it is the only study to demonstrate additive or synergistic effects between HP and growth factors on tissue functional properties. These findings are exciting as coupling HP stimulation with growth factor application has allowed for the formation of tissue engineered constructs with biomechanical and biochemical properties spanning native tissue values.

## Introduction

Hydrostatic pressure plays an important role in the mechanoregulation of several tissues; including cartilage [1]–[8], knee meniscus [9], temporomandibular joint disc [10], [11], intervertebral disc [11]–[13], bone [14], bladder [15], and vasculature [16]. In these studies, HP generally led to increased extracellular matrix (ECM) production. HP application appears particularly promising as a strategy in cartilage tissue engineering, as cartilage degeneration remains a tremendous problem [17]. Following injury, cartilage has a poor ability to self-repair due to its avascularity, and current clinical treatments for articular cartilage injuries result in the formation of mechanically inferior fibrocartilage [18]. Therefore, cartilage regeneration with tissue engineering strategies appears to be a promising approach. A scaffoldless approach to tissue engineering, the self-assembly process, has been developed and utilized by our group to produce engineered constructs with biochemical and biomechanical properties approaching native tissue values [3], [19], [20].

Cartilage is typically exposed to pressures in the physiologic range of 3–18 MPa [21]–[23], and tissue engineering efforts have generally focused on these physiologic pressures. Prior studies have shown complex effects from HP application, demonstrating both inhibition and enhancement of ECM protein production and gene expression depending on the selected HP regimen and culture system. For example, several pioneering studies by Smith et al. [4], [24]–[27] on monolayers have demonstrated enhanced protein production and gene expression when applying intermittent hydrostatic pressure at 10 MPa, 1 Hz to both normal human adult articular chondrocytes as well as to osteoarthritic chondrocytes. However, they found detrimental effects on collagen II mRNA production when applying 10 MPa static (0 Hz) HP to adult articular chondrocytes in monolayer [25]. On the other hand, Mizuno et al. [5] applied 2.8 MPa static HP to 3-D bovine chondrocyte seeded collagen sponges and found an increase in GAG production. Similarly, Toyoda et al. [8], [28] observed significantly increased GAG production, aggrecan mRNA, and type II collagen mRNA expression when applying 5 MPa static HP to bovine articular chondrocyte seeded agarose gels.

Several prior studies have also demonstrated the benefits of growth factors, including BMP-2, IGF-I, and TGF-β1, on construct functional properties [29]–[31]. In recent work (under review, Osteoarthritis and Cartilage), we have demonstrated the benefits of combined BMP-2 and IGF-I treatment on construct compressive properties and GAG production, as well as the benefit of TGF-β1 treatment on construct compressive and tensile properties, with corresponding enhancement of GAG and collagen production. Furthermore, previous work has demonstrated the benefits of combining growth factor application with direct compression mechanical stimulation on construct [32] and explant [33] functional properties.

Though several studies have been performed in assessing the role of HP in tissue biochemistry, to the best of our knowledge, no studies have demonstrated enhanced biomechanical properties from HP application in any tissue. Furthermore, studies that systematically assess the effects of multiple HP magnitudes and frequencies on construct functional properties are lacking. Additionally, there is a dearth of studies demonstrating synergistic effects on tissue functionality from combining hydrostatic pressure and growth factors.

Using a scaffoldless cartilage tissue engineering model [19], [20], this study sought to test the hypotheses that 1) a short-term application of static HP during construct development will have the greatest enhancement of construct biochemical and biomechanical properties, and that 2) there will be additive or synergistic effects when combining growth factors and HP stimulation. These hypotheses were assessed and supported using a two-phased approach. In phase I, a 3×3 full-factorial design of HP magnitude (1, 5, and 10 MPa) and frequency (0, 0.1, and 1 Hz) was used, and the best two treatments were selected for use in phase II. Phase II employed a 3×3 full-factorial design of HP (no HP, 5 MPa static, 10 MPa static) and growth factor application (no GF, BMP-2+IGF-I, TGF-β1) for a total of nine treatment groups.

## Materials And Methods

### Chondrocyte Isolation and Seeding

Cartilage from the distal femur of wk-old male calves was obtained [32], [34], [35] (Research 87, Boston, MA) and digested with collagenase type 2 (Worthington, Lakewood, NJ) for 24 hrs, as described in detail previously [20]. A polysulfone die consisting of 5 mm dia.×10 mm long cylindrical prongs that fit into 6 wells of a 48-well plate was used to construct each agarose mold, as described in detail previously [20]. The culture medium is a chemically defined medium that has been described previously [20]. To each agarose well, 5.5×10^6^ cells were added in 100 μl of culture medium; t = 0 was defined as 24 hrs after seeding.

### Phase I: HP Magnitude and Frequency Selection

At t = 10 days, self-assembled constructs (n = 6/group) were removed from confinement in 5 mm dia. agarose wells and exposed to HP for 1 h/day, for 5 days. The study employed a 3×3 full-factorial design of magnitude (1, 5, 10 MPa) and frequency (0, 0.1, 1 Hz), for a total of 9 treatment groups. The constructs were then placed in individual agarose-coated wells of 48-well culture plates for the remainder of the study. A control (CC) consisted of constructs removed from confinement in 5 mm dia. agarose wells at 10 days, and cultured in individual wells of 48-well culture plates coated with 2% agarose for the remainder of the study. Per construct, 500 μl of medium was changed daily, and all constructs were assessed at t = 4 wks.

### Phase II: Combination of HP and Growth Factors

This study employed a 3×3 full-factorial design of HP (no HP, 5 MPa static, 10 MPa static) and growth factor application (no GF, BMP-2+IGF-I, TGF-β1) for a total of nine treatment groups. The hydrostatic pressure regimens were selected in phase I (please see results), while the growth factor treatments were selected from a prior study by our group (under review, Osteoarthritis and Cartilage). The HP regimens were applied as in phase I, for 1 hr/day, from t = 10–14 days. The specific growth factor treatments were TGF-β1 (30 ng/ml) continuously from t = 0–14 days, or a combined treatment of BMP-2 (10 ng/ml) continuously from t = 10–14 days and IGF-I (10 ng/ml) from t = 0–7 days and t = 14–21 days. All growth factors were obtained from Peprotech Inc. (Rocky Hill, NJ), and applied in the culture medium. As in phase I, constructs were removed from confinement at t = 10 days, and cultured in individual wells for the remainder of the study. Per construct, 500 μl of medium was changed daily, and all constructs were assessed at t = 4 wks.

### Specimen Pressurization

The procedure used has been described previously [3]. Briefly, constructs were placed into heat sealable bags (Kapak/Ampak Flexibles, Cincinnati, OH) with 35 ml medium, and the bags were heat-sealed without any bubbles inside. The chamber was maintained at 37° C during pressurization. Briefly, from t = 10–14 days, the constructs were pressurized at a specific regimen for 1 hr. Following HP application, the pouches were opened with autoclaved instruments and the samples were returned to individual agarose coated wells.

### Histology and Immunohistochemistry

Samples were frozen and sectioned at 14 μm. Safranin-O and fast green staining were used to examine GAG distribution [36], [37]. Picrosirius red was used for qualitative examination of collagen content. A von Kossa stain was used to examine mineralization. IHC was used to determine the presence of collagen types I and II, as described previously [20].

### Quantitative Biochemistry

Samples were frozen overnight and lyophilized for 72 hrs, followed by re-suspension in 0.8 mL of 0.05 M acetic acid with 0.5 M NaCl and 0.1 mL of a 10 mg/mL pepsin solution (Sigma) at 4°C for 72 hrs. Next, 0.1 mL of 10× TBS was added along with 0.1 mL pancreatic elastase and mixed at 4°C overnight. From this digest, total DNA content was measured by Picogreen® Cell Proliferation Assay Kit (Molecular Probes, Eugene, OR). Total sulfated GAG was quantified using the Blyscan Glycosaminoglycan Assay kit (Biocolor) [38], [39]. Total collagen content was assessed by a chloramine-T hydroxyproline assay [40].

### Mechanical Testing

To obtain salient compressive properties, samples were evaluated under conditions of creep indentation [41], which has been described in detail previously [20]. The aggregate modulus (H_A_), permeability, and Poisson's ratio of the samples were then determined using the linear biphasic theory [42]. To obtain construct tensile properties, uniaxial tests were run on a materials testing system (Instron Model 5565, Canton, MA) with a 50 N load cell, as described previously [43]. Stress-strain curves were created from the load-displacement curve and the cross-sectional area of each sample, and Young's modulus (E_Y_) was calculated from the linear region of each stress-strain curve. Construct thickness was measured using digital calipers.

### Statistical Analysis

Biochemical and biomechanical assessments were performed on all constructs (n = 6 or 7). In each phase, a single factor ANOVA was used to analyze the samples, and a Fisher LSD post hoc test was used when warranted. Significance was defined as p<0.05. Additionally, in phase II, the interaction term of a two factor ANOVA was used to test for synergism, as described previously [44], with significance defined as p<0.05.

## Results

### Gross Appearance and Histology

**Figure 1:**
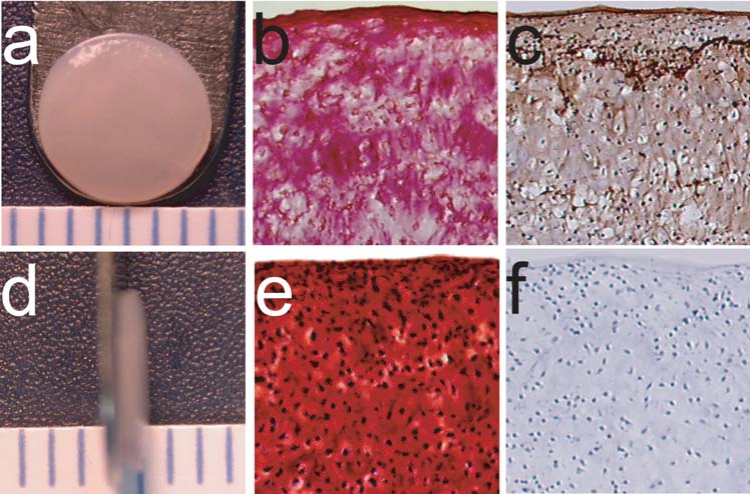
Histological and immunohistochemical images representative of all self-assembled constructs (10x original magnification). (a) Gross morphology. (b) Picrosirius red stained sections. (c) Collagen II IHC sections. (d) Gross morphology profile. (e) Safranin-O/fast green stained sections. (f) Collagen I IHC sections.

All constructs reached a diameter of approximately 6 mm at t = 4 wks (Fig. 1a). In phase I, there were no differences in wet weight (WW) or thickness among the treatment groups, as demonstrated in Table 1. However, as shown in Table 2, in phase II, there was a decrease in construct WW and thickness in all groups treated with TGF-β1.

**Table 1:** Phase I construct properties.

Group	WW (mg)	Thickness (mm)	H_A_ (kPa)	E_Y_ (kPa)	GAG/WW (%)	Col./WW (%)	Total Cells (×10^6^)
**Control**	17.1±1.7	0.70±0.07	138±30	596±185	6.2±0.3	7.1±1.8	4.7±0.9
**1 MPa, 0 Hz**	17.1±1.2	0.65±0.05	268±45	739±252	6.7±0.5	7.1±2.3	5.3±0.5
**1 MPa, 0.1 Hz**	17.2±1.8	0.69±0.08	180±33	508±176	7.2±0.5	8.4±1.4	5.3±0.4
**1 MPa, 1 Hz**	17.7±1.0	0.78±0.09	196±104	871±221	6.0±0.6	8.2±1.2	4.9±1.3
**5 MPa, 0 Hz**	15.2±2.8	0.67±0.08	269±44	1196±271	8.1±0.6	9.4±2.5	4.4±0.6
**5 MPa, 0.1 Hz**	17.4±1.5	0.70±0.06	210±84	837±148	6.9±0.4	6.7±0.6	4.8±0.5
**5 MPa, 1 Hz**	16.1±1.2	0.67±0.14	181±94	708±103	6.2±1.6	6.5±1.0	4.5±0.5
**10 MPa, 0 Hz**	14.4±2.2	0.67±0.05	270±46	1144±281	8.1±0.4	10.8±1.9	4.3±0.3
**10 MPa, 0.1 Hz**	16.6±2.8	0.67±0.06	208±42	958±154	7.4±0.5	6.1±2.2	4.9±0.5
**10 MPa, 1 Hz**	16.9±3.5	0.64±0.05	287±82	935±221	9.1±0.8	6.0±1.7	4.2±0.4

Col., total collagen

**Table 2:** Phase II construct properties.

Group	WW (mg)	Thickness (mm)	H_A_ (kPa)	E_Y_ (kPa)	GAG/WW (%)	Col./WW (%)	Total Cells (×10^6^)
**No HP, No GF**	32.1±0.7	0.98±0.09	94±24	619±73	5.2±0.5	5.6±1.5	5.0±0.5
**No HP, BMP-2+IGF-I**	33.0±1.8	1.09±0.11	160±29	596±70	6.9±1.3	5.4±1.4	5.1±1.5
**No HP, TGF-β1**	16.2±1.1	0.69±0.06	176±38	1460±182	7.3±0.3	9.2±2.0	5.0±4.5
**5 MPa, No GF**	29.0±1.7	1.01±0.15	173±87	1424±465	7.8±0.6	7.5±0.5	5.7±0.3
**5 MPa, BMP-2+IGF-I**	32.0±2.5	1.03±0.14	165±37	862±293	7.4±0.8	5.8±0.4	5.2±0.5
**5 MPa, TGF-β1**	15.4±1.0	0.65±0.06	189±46	1545±235	8.1±0.2	12.6±2.4	5.1±0.4
**10 MPa, No GF**	27.8±0.8	0.94±0.13	161±19	1268±404	8.5±0.6	7.8±1.5	5.6±0.4
**10 MPa, BMP-2+IGF-I**	31.4±1.3	1.06±0.09	187±45	776±260	7.5±0.4	5.6±1.2	5.6±0.1
**10 MPa, TGF-β1**	14.8±0.4	0.69±0.08	248±37	2048±266	9.6±0.4	15.3±2.9	5.5±0.4

Col., total collagen

In both studies, positive staining for collagen (Fig. 1b) and GAG (Fig. 1e) was observed throughout the construct thickness. Additionally, based on IHC, collagen II was expressed throughout each construct (Fig. 1c), with no collagen I production (Fig. 1f). Finally, in phase II, there was no mineralization or chondrocyte hypertrophy observed with BMP-2+IGF-I treatment.

### Quantitative Biochemistry

**Figure 2:**
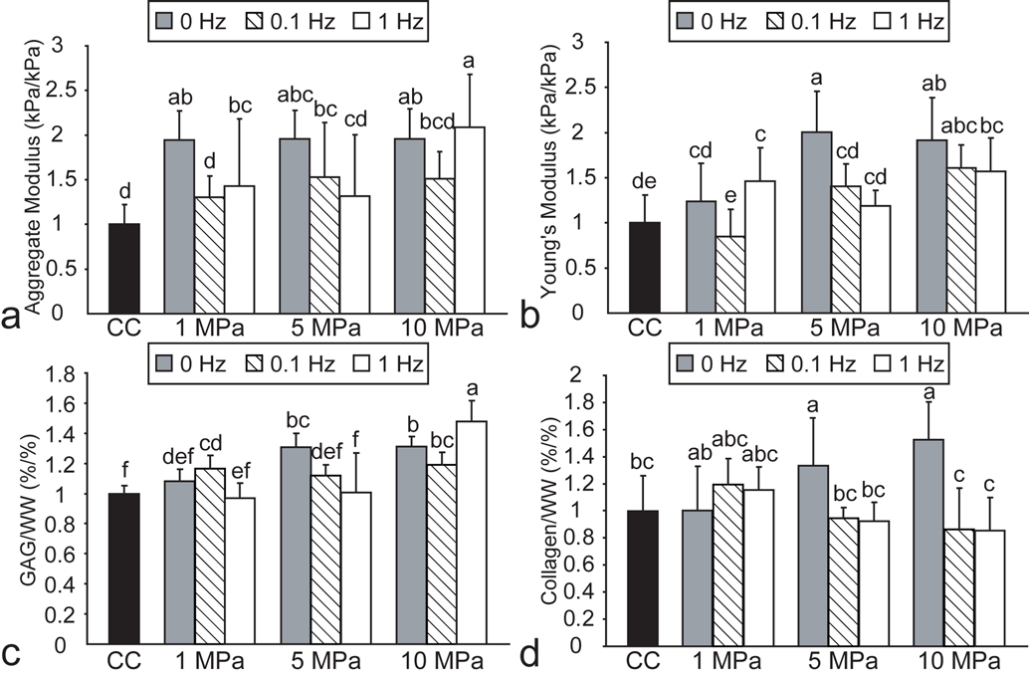
Biomechanical and biochemical properties of self-assembled constructs in phase I, normalized to control values. HP application at 5 or 10 MPa, 0 Hz, resulted in a significantly higher (a) aggregate modulus, (b) Young's modulus, (c) GAG/WW and (d) collagen/WW than control. Columns and error bars represent means and standard deviations. Groups denoted by different letters are significantly different (p<0.05).

In phase I, all values of cells/construct, GAG/WW, and collagen/WW are found in Table 1. There were no differences in cells/construct among the different treatment groups. Several treatments resulted in significant increases in GAG/WW, but the greatest increases in GAG/WW were observed with the 5 MPa static, 10 MPa static, and 10 MPa, 1 Hz regimens (Fig. 2c), with GAG/WW values of 8.1±0.6, 8.1±0.4, and 9.1±0.8%, respectively. However, only 5 MPa static and 10 MPa static HP application significantly increased collagen/WW (Fig. 2d), with values of 9.4±2.5 and 10.8±1.9%, respectively.

**Figure 3:**
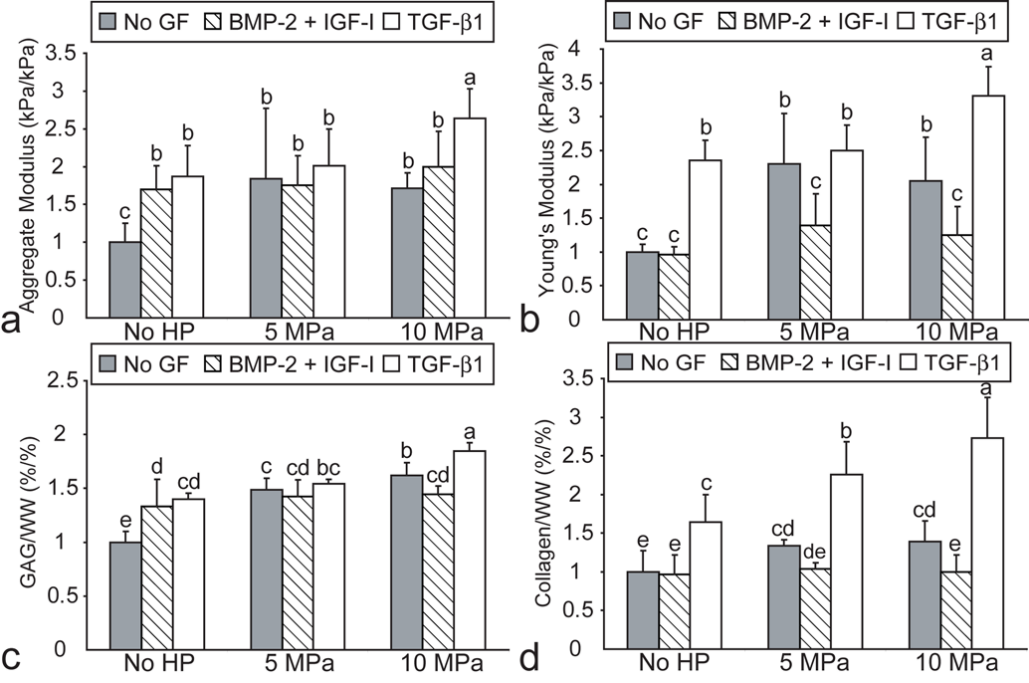
Biomechanical and biochemical properties of self-assembled constructs in phase II, normalized to control values. (a) aggregate modulus, (b) Young's modulus, (c) GAG/WW and (d) collagen/WW. Combined treatment with 10 MPa static HP and TGF-β1 led to additive increases in aggregate modulus and Young's modulus, and a synergistic increase in collagen/WW. Columns and error bars represent means and standard deviations. Groups denoted by different letters are significantly different (p<0.05).

In phase II, all values of cells/construct, GAG/WW, and collagen/WW are found in Table 2. There were no differences in cells/construct among the different treatment groups. All treatments exhibited a significant increase in GAG/WW (Fig. 3c); additionally, there was an adjunctive effect between 10 MPa static HP and TGF-β1, as their combination resulted in a greater GAG/WW, of 9.6±0.4%, than either treatment alone. Treatment with either HP regimen or with TGF-β1 significantly increased the collagen/WW (Fig. 2d). Furthermore, combined treatment with 10 MPa static HP and TGF-β1 led to a synergistic increase in collagen/WW to 15.3±2.9%; the increase in collagen/WW was statistically greater than the sum of either treatment alone.

### Mechanical Evaluation

In phase I, all values of H_A_ and E_Y_ are found in Table 1. The 1, 5, and 10 MPa static HP groups, as well as the 10 MPa, 1 Hz group all demonstrated a significant increase in H_A_ relative to the control group (Fig. 2a), with values of 268±45, 269±44, 270±46, and 287±82 kPa, respectively. However, only the 5 MPa static HP group exhibited significant increases in E_Y_ to 1196±271 kPa (Fig. 2b); a similar increase in E_Y_ to 1144±281 kPa was observed for the 10 MPa static HP group.

In phase II, all values of H_A_ and E_Y_ are found in Table 2. All treatments exhibited a significant increase in H_A_ (Fig. 3a), with the 10 MPa+TGF-β1 treatment group displaying the greatest increase, to 248±37 kPa. This increase indicated an additive effect between 10 MPa static HP and TGF-β1, as the effect of their combined use on H_A_ was equal to the sum of the effects of either treatment alone. Treatment with either HP regimen alone or with TGF-β1 significantly increased the E_Y_; furthermore, combined treatment of 10 MPa static HP and TGF-β1 led to an additive increase in E_Y_ to 2048±266 kPa (Fig. 3b).

## Discussion

This study employed a 2-phased approach to choose an optimal HP loading regimen, as well as to determine the effects of combined growth factor and HP application. To the best of our knowledge, this study is the first to 1) demonstrate increases in the biomechanical properties of tissue from pure HP application, using a cartilage model, 2) demonstrate additive or synergistic effects between HP and growth factors on tissue functional properties, and 3) systematically assess the effects of varying physiologic magnitudes and frequencies of HP on tissue functional properties.

In phase I, 5 MPa and 10 MPa static HP were the only regimens that increased both H_A_ and E_Y_ with parallel increases in GAG and collagen content. These results support our hypothesis, as static hydrostatic pressure was found to have the greatest effect on construct biochemical and biomechanical properties. Since 5 MPa and 10 MPa static HP were the only regimens to significantly increase the compressive and tensile stiffness as well as GAG/WW and collagen/WW, these two regimens were selected for use in phase II.

In phase II, the combination of 10 MPa static HP and TGF-β1 treatment had significant effects on construct biomechanical and biochemical properties, thus supporting the hypothesis that combined HP and growth factor treatment would have additive and synergistic effects on construct functional properties. The combined treatment of 10 MPa static HP and TGF-β1 had an additive effect on both H_A_ and E_Y_, as the increases in compressive and tensile stiffness for the combined treatment were equal to the sum of the effects of the two individual treatments. Additionally, the combined treatment exhibited a synergistic increase in collagen/WW, as the effect of the combined treatment was statistically greater than the sum of the effects of each individual treatment. Excitingly, the collagen/WW of this group, at 15.3%, spanned reported values for native articular cartilage [45].

However, although 5 MPa and 10 MPa static HP have similar effects on construct properties when applied alone, 5 MPa static HP did not exhibit the same additive and synergistic effects when combined with TGF-β1 treatment. This result suggests that there are different cellular responses to varying HP magnitudes; for example, it can be speculated that increasing HP from 5 MPa to 10 MPa in the presence of TGF-β1 may activate additional intracellular pathways that lead to enhanced production of ECM proteins and increased biomechanical properties. Interestingly, a similar effect has been observed previously in work on chondrogenic differentiation of human mesenchymal stem cells (MSCs) [46]. It was found that collagen II mRNA expression of MSCs cultured with TGF-β3 responded maximally to 10 MPa HP application.

It is also interesting to note that combining BMP-2+IGF-I treatment with either of the HP treatments did not lead to further enhancement of construct properties, and actually negated the beneficial effects of HP alone on construct properties. It has previously been shown that HP modulates the level of TGF-β mRNA [47]. Additionally, combined treatment with TGF-β1 and IGF-I has detrimental effects on GAG and collagen content shown by Blunk et al. [29] and our own work (under review, Osteoarthritis and Cartilage). Based on these prior studies, one can speculate that HP application may lead to the production of TGF-β1, which, when combined with the effects of exogenously applied IGF-I may have detrimental effects, as seen previously, although it is possible that enhanced TGF-β1 mRNA expression may not correspond to increased TGF-β1 production due to the extensive post-transcriptional and post-translational regulation of TGF-β1, as reviewed previously [48]. In future studies, it would be exciting to elucidate the pathways involved in HP signal transduction, and how they coincide with the growth factor signal transduction cascades. Since the exact pathways for HP signal transduction have not been elucidated, we can only speculate that the pathways leading to increased matrix synthesis are either further enhanced, when combining HP and TGF-β1, or perhaps inhibited, when combining HP and the BMP-2+IGF-I combination.

By demonstrating the beneficial effects of static HP over cyclic HP application on construct biomechanical and biochemical properties, this study contradicts several prior studies that have shown positive effects from cyclic HP [4], [7], [24]–[27]. Though when comparing these studies, it is important to note that HP was applied to chondrocytes in monolayer rather than 3-D constructs. Furthermore, these studies utilized adult or osteoarthritic chondrocytes which behave substantially differently than the immature bovine chondrocytes used in this study [49]. On the other hand, the results of this study agree with the conclusions of several other studies that applied static HP to 3-D constructs and found beneficial effects on construct biochemical properties [5], [8], [28].

When assessing the effects of combined HP and growth factor treatment on cartilage properties, the results presented here agree with prior studies that have combined these treatments as differentiation agents for mesenchymal stem cells [46], [50]. For example, Miyanishi et al. [46] found that combined HP application with TGF-β3 increased SOX9, collagen II, and aggrecan mRNA levels 1.9, 3.3, and 1.6-fold, respectively, more than treatment with TGF-β3 alone. It is also known that another form of mechanical stimulation, namely direct compression, exhibits synergistic effects when combined with growth factor treatment on articular cartilage constructs [32] and explants [33]. Specifically, Mauck et al. [32] found that combined treatment with dynamic compression and TGF-β1 resulted in a 277% increase in equilibrium aggregate modulus, while Bonassar et al. [33] observed a 290% increase in proteoglycan synthesis with combined dynamic compression and IGF-I treatment.

Physiologic HP does not deform cartilage [51]; therefore, the enhanced construct biomechanical properties observed in this study must be accounted for by other mechanisms. As reviewed elsewhere [52], on the microscopic level, HP can compress void spaces within and around proteins on the cell surface. At a certain pressure, the compression of void spaces becomes great enough that the protein can achieve a lower energy state by changing its conformation. Cell surface ion channels may serve as “pressure sensors,” altering their conformations and thus changing the intracellular ion concentrations depending on the applied pressure. For example, Hall [1] found that in chondrocytes, the activity of the Na/K pump was suppressed substantially with 10 MPa static HP application for 10 min, while the Na/K/2Cl transporter was more sensitive to HP application. Also, Browning et al. [53] observed activation of the Na/H pump in bovine articular chondrocytes with HP application at approximately 10 MPa. Additionally, Mizuno [54] found that HP increases intracellular calcium through the activation of stretch-activated channels. Since protein synthesis is affected by intracellular ion concentrations [55], it is envisioned that different ion channel responses to varying HP magnitudes alters the intracellular ion flux and stimulates signal transduction cascades for upregulation of ECM-specific genes, enhanced ECM protein production, and increased biomechanical properties as observed in this study. Growth factors may serve as an adjunctive method for stimulating similar downstream pathways, thus leading to additive and synergistic effects, as observed in this study.

The beneficial effects of HP on tissue biochemical properties are not confined merely to cartilage, and it is possible that the approach of this study, namely combining optimized HP and growth factor treatments, may be applicable to several other tissues. For example, Stover et al. [15] found that applying cyclic HP to bladder smooth muscle cells resulted in a proliferative response suggestive of tissue remodeling. Also, Reza and Nicoll [12] observed increased production of collagen II in intervertebral disc cells from the outer annulus exposed to 5 MPa HP. Additionally, Almarza and Athanasiou [10] demonstrated increased collagen I gene expression and protein production when applying 10 MPa static HP to temporomandibular joint disc cells. Finally, Suzuki et al. [9] applied 4 MPa static HP to knee meniscal cells, and found a significant increase in collagen I mRNA and a significant decrease in matrix metalloproteinase -1, and -13. Although none of these studies assessed the effects of HP on biomechanical properties, it can be speculated that coupling these HP regimens with the application of exogenous bioactive agents specific to these tissues, may also result in additive and synergistic effects on the functional properties.

Multiple studies have assessed the effects of both static and intermittent HP regimens on gene expression and protein production. This study, which investigated the effects of multiple HP magnitudes and frequencies on construct functional properties, demonstrated enhanced biomechanical and biochemical tissue properties. Additionally, it systematically assessed the effects of combining HP and growth factors on construct functional properties, and indicated synergistic and additive effects. Future studies should determine the effects of temporal HP application during construct development, as well as examine the immediate and long-term effects of HP application on construct properties.
